# Ecological multivariate assisted spectrophotometric methods for determination of antipyrine and benzocaine HCl in presence of antipyrine official impurity and benzocaine HCl degradant: toward greenness and whiteness

**DOI:** 10.1186/s13065-024-01352-7

**Published:** 2024-12-24

**Authors:** Khadiga M. Kelani, Maha A. Hegazy, Amal M. Hassan, Ahmed H. Nadim

**Affiliations:** 1 Pharmaceutical Analytical Chemistry Department, Faculty of Pharmacy, Modern University for Technology and Information, El-Hadaba El-Wosta, Mokatam, 5th district, Cairo, Egypt; 2 Pharmaceutical Analytical Chemistry Department, Faculty of Pharmacy, Cairo University, Cairo, Egypt; 3 Pharmaceutical Analytical Chemistry Department, Faculty of Pharmacy, Future University in Egypt, 90th St, New Cairo 3, Cairo, Egypt

**Keywords:** Antipyrine, Benzocaine HCl, Partial least squares, Artificial neural network, Green analytical chemistry, Multivariate curve resolution-alternating least squares

## Abstract

A simple and green chemometrics-assisted spectrophotometric technique has beendeveloped and validated for the determination of antipyrine (ANT) and benzocaine HCl (BEN) along with the official impurity of ANT, antipyrine impurity A (ANT imp-A), and the degradation product of BEN, p-amino benzoic acid (PABA), in their quaternary mixture. Three models were developed and compared: partial least squares (PLS), artificial neural networks (ANN), and multivariate curve resolution-alternating least squares (MCR-ALS) where the four studied drugs were successfully quantified. The quantitative determination of the studied drugs was assessed using percentage recoveries, standard errors of prediction, and root mean square errors of prediction. The ANN model demonstrated the lowest error and the best correlation making it the most accurate method for analysis. The models were constructed in the ranges of 5.0–9.0 µg mL^−1^ for ANT, 1.0–5.0 µg mL^−1^ for BEN, 0.5–2.5 µg mL^−1^ for ANT imp-A, and 0.25–1.25 µg mL^−1^ for PABA. The established models successfully determined ANT, BEN, ANT imp-A, and PABA with detection limits of 0.312, 0.178, 0.093, and 0.042 µg mL^−1^ for PLS, 0.185, 0.085, 0.001, and 0.034 µg mL^−1^ for ANN; and 0.473, 0.240, 0.073, and 0.069 µg mL^−1^ for MCR-ALS, respectively. The greenness and the whiteness of the proposed method were assessed using two green evaluating approaches: analytical Eco-scale, and AGREE, along with one white analytical chemistry evaluating tool, RGB. The three proposed models were successfully applied for determination of ANT and BEN in their pharmaceutically co-formulated dosage forms. They are also recommended for stability assays and purity testing of these drugs in quality control laboratories.

## Introduction

One of the critical steps in pharmaceutical analysis is the purity assessment of the active pharmaceutical ingredients to ensure their safety and quality. The identification and quantification of impurities in either bulk drug or dosage form is a concern in the analytical platform [1]. Impurities could originate from several sources such as degradation products, excipients, and during manufacturing steps [2]. Therefore, the development of analytical techniques that could determine the pharmaceutical drug along with its impurities is considered vital [3]. Spectrophotometric methods have the advantages of being simple and economical compared to other analytical techniques. However, the presence of multi-ingredient formulations, along with their impurities or degradation products exhibiting overlapping spectra would hinder their determination using conventional spectrophotometric methods. Considering that incorporating more informative variables in model construction enhances accuracy and robustness, it is essential to adopt advanced analytical approaches. Thus, in such cases, multivariate data analysis is the optimum choice for handling these complex matrices, as it outperforms traditional univariate methods [4–6]. Chemometric techniques had facilitated the mathematical resolution of overlapped spectra with minimum background noise interference based on their mathematical and statistical abilities. There are different algorithms of the chemometric techniques that have been established, and from these chemometric methods three models were selected to perform our study, namely; partial least squares (PLS), artificial neural networks (ANN), and multivariate curve resolution-alternating least squares (MCR-ALS).

The PLS model was selected due to its widespread use as a multivariate method, capable of establishing optimum correlation between the concentration levels and the analyzed spectra, and to reach the highest variance. In addition, ANN was chosen as an advanced technique with superior characteristics to improve the concentration profile of the target analytes. Furthermore, MCR-ALS model was selected as an advanced technique that enables not only the quantitative analysis of the studied drugs but also qualitative analysis, thereby enriching their determination [7, 8].

**Fig. 1:**
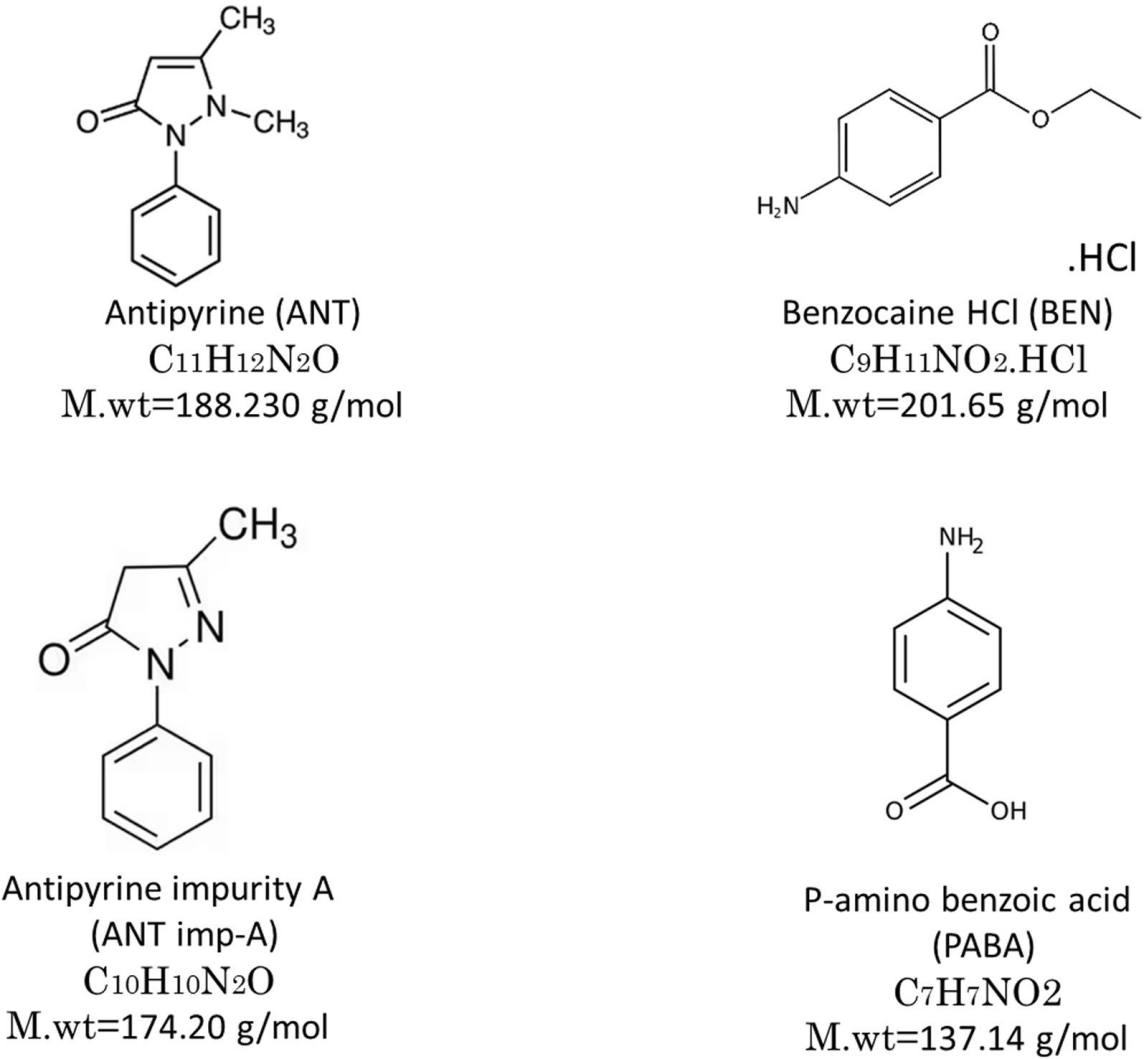
Chemical structures of the four cited components

Antipyrine (ANT), 21,5-dimethyl-2-phenyl-4-pyrazolin-3-one, is a nonsteroidal anti-inflammatory drug (Fig. 1) [9, 10]. Reviewing its pharmacopeial monographs [11–13] revealed that its determination was carried out using titration methods. In contrast, only one impurity, identified as antipyrine impurity A (ANT imp-A), was reported in the European Pharmacopoeia (Ph. Eur.). A literature review showed that ANT has been quantified either alone or in combination with other compounds using spectrophotometric [10, 14–17], chromatographic [18–23] and electrochemical methods [9, 24]. Benzocaine HCl (BEN), 4-aminobenzoic ethyl ester in HCl, is a local anesthetic that acts on different mucous membranes (Fig. 1) [9, 10]. It is an official drug listed in both the British pharmacopeia (BP) and United States pharmacopeia (USP) [11, 12]. Previous reports have shown that BEN has been quantified either alone or in presence of other drugs using various techniques, including spectrophotometric methods [10, 25, 26], HPLC [22, 23, 27–30] and electrochemical methods [9, 31, 32].

The combination of ANT and BEN in ear drops has been shown to be more effective in treatment of ear inflammatory disorders, such as otitis media and otitis externa, compared to using each drug alone [22]. Literature review indicated that ANT and BEN have been determined using spectrophotometric [10], chromatographic [22, 23] and electrochemical techniques [9]. However, none of the previously reported methods considered the simultaneous determination of ANT and BEN in presence of the official impurity of ANT (ANT imp-A) and the primary degradation product of BEN, p-amino benzoic acid (PABA). To the best of our knowledge, no multivariate spectrophotometric method had been reported for determination of these compounds.

Green analytical chemistry (GAC) has recently gained sufficient attention in the analytical field. The use of less toxic, bio-accumulative and hazardous solvents or reducing the generation of waste products would have positive impact on human health and the environment [33]. Several approaches were used to evaluate methods’ greenness, including analytical Eco-scale [34] and the recently developed approach, the Analytical Greenness tool (AGREE) [35]. In addition, the principles of GAC were extended to a new concept of white analytical chemistry (WAC). In WAC, a Red–Green–Blue (RGB) model is developed covering the quality of the analytical method (red), method efficiency and the practical criteria (blue), in addition to green aspects (green) [36].

The objective of this work is to provide three chemometrics-assisted spectrophotometric models, namely, PLS, ANN and MCR-ALS for the quantification of the co-formulated drugs ANT and BEN along with ANT impurity A (ANT imp-A) and acidic degradation product of BEN, PABA, for the first time. Additionally, the greenness and whiteness profile of the proposed methods were assessed. The proposed methods offer the advantages of being green, simple, cost-effective, while successfully determining the four cited compounds both in their pure forms and in pharmaceutical dosage forms.

## Methods

### Instruments and software

Measurements were carried out using Shimadzu UV 1601 dual beam spectrophotometer (Kyoto, Japan). Spectra scanning were conducted at 200.0–400.0 nm range with 0.2 nm intervals using 1.00-cm quartz cuvettes. All the proposed models were determined using Matlab® software (7.0.1). MCR-ALS needed an extra toolbox downloaded from (http://www.mcrals.info) [37].

### Materials

#### Chemicals and materials

ANT and BEN standards were purchased from Thermo Fisher Scientific (USA). Their potencies were evaluated according to the reported method and found to be 100.03 ± 0.961% and 99.08 ± 0.854%, respectively [22]. ANT imp-A (99.04 ± 0.721%) and PABA (99.82 ± 0. 566%) were purchased from Sigma-Aldrich (Germany). Ear calm® ear drop was the product of Riyadh pharma, medical & cosmetic products Co, Saudi Arabia (batch no. 23DB19), labeled to contain 50.0 mg ANT and 10.0 mg BEN in 1 mL distilled water was obtained from Otsuka (Egypt).

#### Standard solutions

Four standard solutions of ANT, BEN, ANT imp-A and PABA were individually prepared, by accurately weighing 10.0 mg of each compound into separate 100-mL volumetric flasks, using distilled water as diluent to obtain a final concentration of 0.1 mg mL^−1^.

### Procedures

#### Construction of calibration models

Brereton five-level four-factor design was followed [38]. Spectral features of the four studied drugs were assessed by measuring solutions of various concentrations against distilled water as blank, over the wavelength range 200.0–400.0 nm. Twenty-five samples were prepared to contain different concentrations of ANT, BEN, ANT imp-A and PABA in the ranges of 5.0–9.0, 1.0–5.0, 0.5–2.5 and 0.25–1.25 µg mL^−1^, respectively. Samples were prepared by transferring different aliquots of the four cited components from their standard solutions in sets of 10-mL volumetric flasks and the volume was completed using distilled water. The solutions were then scanned over the wavelength range 200.0–400.0 nm at 0.2 intervals, and the spectra were recorded. The range from 250.0 nm to 315.0 nm with 326 experimental points were transferred to Matlab® for further analysis. Then, the multivariate calibration models were developed using fourteen of the previously prepared samples. Prior to calibration, all the spectra were mean-centered for the construction of the PLS, ANN, and MCR-ALS models.

#### Validation of the calibrated models

The remaining eleven samples from the previously prepared mixtures were used for validation. The parameters obtained from PLS, ANN, and MCR-ALS were used for the quantification of the four cited components.

#### Application to Ear calm® ear drops

A 1.0-mL aliquot was transferred from Ear calm® ear drops to a 100-mL flask, and 50.0 mL distilled water was added. The flask was sonicated for 10.0 min and then diluted to the mark using distilled water. Then, 1.0-mL aliquot of the previously prepared flask was transferred to 100-mL flask and diluted with distilled water to obtain a final concentration of 5.0 µg mL^−1^ for ANT and 1.0 µg mL^−1^ for BEN. The prepared solutions were scanned and the concentrations were predicted using the developed models.

## Results and discussion

**Fig. 2:**
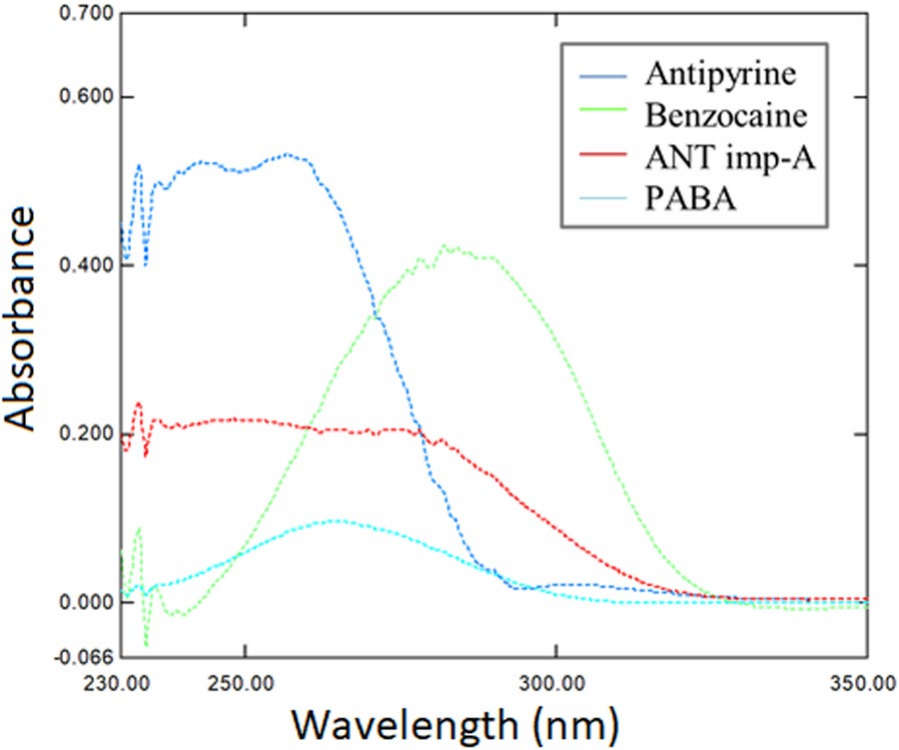
Normalized spectra of 10.0 μg mL^−1^ ANT, 5.0 μg mL^−1^ BEN, 5.0 μg mL^−1^ ANT imp-A and 1.0 μg mL^−1^ PABA using distilled water as diluent

Spectrophotometry is regarded as a reliable and economical method due to its ability to resolve overlapping spectra using various techniques. Consequently, it is one of the most widely used methods for the determination of multi-component pharmaceutical dosage forms. The technique offers several advantages including, inexpensive instrumentation, no requirement for sample pretreatment, and being a green, safe, and less hazardous option to the environment [4]. The multivariate technique is one of the most valuable methods for resolving severe and complete overlapping. As shown in Fig. 2, severe overlapping was observed between the spectra of the four studied compounds ANT, BEN, ANT imp-A and PABA. Additionally, the similarity between the spectra of ANT and its impurity presents a significant challenge for classical spectrophotometric techniques. Therefore, integrating multivariate calibration models with spectrophotometry can effectively resolve such complex spectral data.

### Calibration and validation

The three selected and constructed models were PLS, ANN, and MCR-ALS. Absorbance data in the range of 250.0–315.0 nm for the 25 mixtures were chosen for data analysis and calculation. Absorbance below 250.0 nm was disregarded due to high noise that could affect the results, while absorbance above 315.0 nm was also excluded as the four cited compounds exhibited almost zero absorption, making them less informative. Twenty five mixtures of the four cited compounds were prepared following Brereton’s five-level calibration design [39]. The mixtures were divided into two groups: 14 mixtures were used to construct the calibration model, while the remaining 11 mixtures served as an external validation set, as shown in Table 1. It is important to note that a limitation in the analyzed concentration range occurred due to the high absorption of ANT and BEN. According to the reported methods [10] and as shown in Fig. 2, using only 10.0 µg mL^−1^ from ANT and BEN would exceed 1.5 Absorbance units in the spectrum. Thus, adding the analyzed impurities will further increase the absorbance, potentially leading to appearance of noise. Moreover, the selected ranges were within the linearity range of each compound.

**Table 1:**
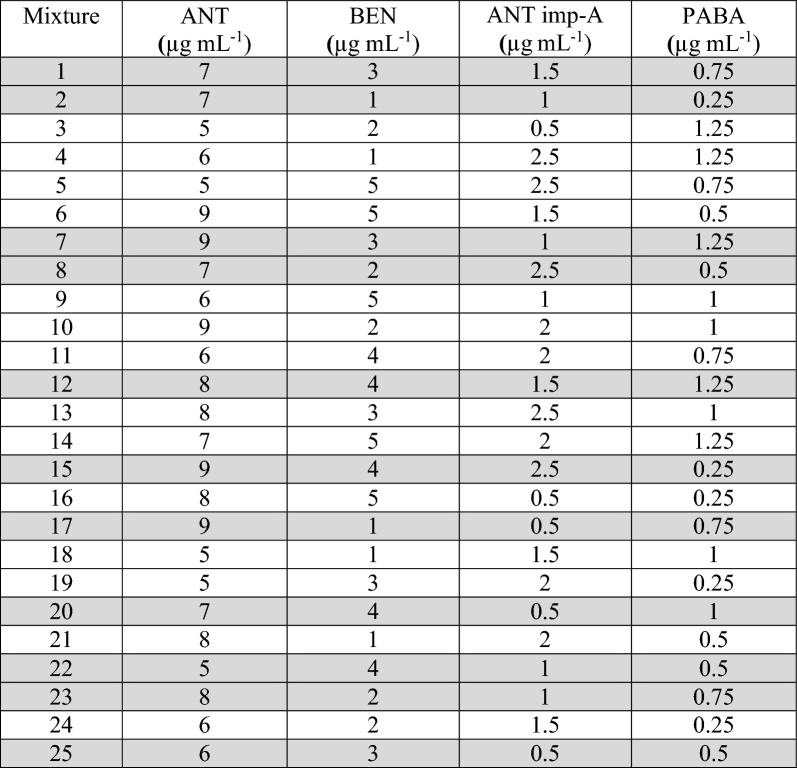
Concentrations of the calibration and validation sets for the four cited compounds

The shaded rows indicate the validation set

#### Partial least squares (PLS) model

**Fig. 3:**
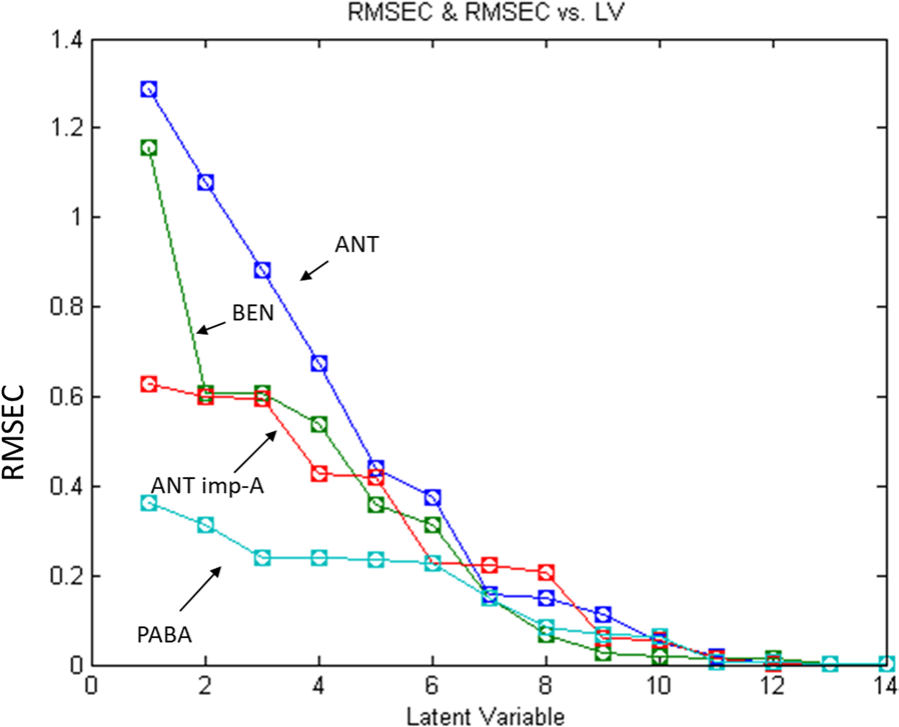
Root mean square error of calibration (RMSEC) versus the number of latent variables used to construct the PLS calibration for the assay of ANT, BEN, ANT imp-A and PABA in their mixtures

The PLS approach is one of the simplest and most widely used techniques to construct multivariate models based on arranging the data into blocks or matrices. In PLS, rather than identifying hyperplanes of maximum variance between the response and independent variables, it constructs a linear regression model by projecting the predicted variables and the observable variables into a new space. Due to projecting the X and Y data to new spaces, the PLS model is also known as the bilinear factor model. PLS composes the spectral and concentration matrices using the following equations:

$${\text{X }} = {\text{ TP }} + {\text{ E}}$$

$${\text{Y }} = {\text{ UQ }} + {\text{ E}}$$

where T and U are the scores of X and Y, respectively. P and Q are the loadings of X and Y, respectively. Mathematical calculation involves the use of columns in the Y matrix to calculate the factors in X; at the same time, the columns of X are used to calculate the factors in Y. This approach represented the matrices by unobservable or “latent” variables [40, 41]. The latent variables can also be defined as the formed new dimension for the analyzed spectral data matrix of the calibration model based on the information obtained from the different values of each analyzed drug concentration. The PLS model was developed by using mean centering as a preprocessing step and leaving one out as a cross-validation by eliminating only one component at a time to obtain the optimum latent variables’ number (LVs), as it should be carefully adjusted to prevent model over fitting [7, 42]. The selected 7 latent variables (LVs) were determined to be the optimum number based on the calculated F test (Fig. 3). Figure 3 shows the relation between LVs and the root mean square error of calibration (RMSEC). The predictive ability of the PLS model was assessed using the validation set mixtures. In Table 2, the mean recovery percentages, SDs % and the specificities (RSD%) were recorded for each drug. Table 3 shows the linearity parameters as well as limits of detection (LOD) and quantification (LOQ), along with the standard error of prediction (SEP) and root mean square error of prediction (RMSEP).

**Table 2:** Prediction recoveries of the validation set samples by the constructed PLS, ANN and MCR-ALS models

Mix no	ANT				BEN				ANT imp-A				PABA			
	Actual conc µg mL^−1^	Prediction recoveries ^a^			Actual conc µg mL^−1^	Prediction recoveries^a^			Actual conc µg mL^−1^	Prediction recoveries^a^			Actual conc µg mL^−1^	Prediction recoveries^a^		
		PLS	ANN	MCR-ALS		PLS	ANN	MCR-ALS		PLS	ANN	MCR-ALS		PLS	ANN	MCR-ALS
1	7.0	98.26	99.98	101.29	3.0	103.73	98.66	102.35	1.5	103.49	99.97	100.15	0.75	102.26	101.66	104.95
2	7.0	97.15	100.02	103.63	1.0	101.45	98.78	100.85	1.0	99.97	99.95	103.77	0.25	101.52	101.10	101.76
3	9.0	97.63	101.00	97.53	3.0	102.64	101.41	99.59	1.0	100.82	100.07	103.10	1.25	97.02	99.88	95.98
4	7.0	99.08	99.99	101.16	2.0	102.22	101.04	102.84	2.5	103.00	100.02	103.30	0.5	98.47	98.66	100.42
5	8.0	97.37	100.02	101.76	4.0	98.29	99.68	103.84	1.5	99.96	99.97	99.99	1.25	99.25	101.25	100.73
6	9.0	98.85	101.00	101.63	4.0	99.28	99.95	101.45	2.5	98.44	99.99	101.08	0.25	99.93	101.22	103.46
7	9.0	97.03	102.02	99.82	1.0	97.24	101.18	98.10	0.5	100.09	99.98	97.12	0.75	97.27	99.09	98.68
8	7.0	100.93	99.97	98.54	4.0	101.84	99.39	100.38	0.5	98.67	99.98	98.94	1.0	98.61	101.80	97.16
9	5.0	102.87	100.04	96.70	4.0	100.42	100.42	102.83	1.0	102.39	99.99	102.62	0.5	101.08	97.80	101.30
10	8.0	96.72	98.96	101.65	2.0	101.70	98.59	98.89	1.0	100.58	100.00	98.15	0.75	100.48	102.60	100.16
11	6.0	99.35	99.99	99.11	3.0	99.69	100.85	98.17	0.5	99.90	100.10	102.14	0.5	97.65	98.00	97.28
Mean ± SD		98.66 ± 1.875	100.27 ± 0.797	100.26 ± 2.095		100.77 ± 1.974	99.99 ± 1.054	100.84 ± 1.996		100.66 ± 1.651	100.00 ± 0.045	100.94 ± 2.245		99.41 ± 1.781	100.28 ± 1.663	100.17 ± 2.738
Specificity (RSD %)		1.900	0.795	2.090		1.959	1.054	1.973		1.640	0.045	2.224		1.792	1.658	2.733

^a^The prediction recovery = predicted concentration/actual concentration × 100

**Table 3:** Regression parameters and model characteristics of the validation sets using the proposed models

Component	Model	Slope	Intercept	R	LOD^a^ (µg mL^−1^)	LOQ^a^ (µg mL^−1^)	SEP^b^	RMSEP^b^
ANT	PLS	0.9217	0.4679	0.9973	0.312	0.945	0.190230	0.172070
	ANN	1.0306	− 0.2028	0.9991	0.185	0.561	0.079014	0.071471
	MCR-ALS	1.0205	− 0.1269	0.9939	0.473	1.432	0.159194	0.143997
BEN	PLS	0.9989	0.0250	0.9989	0.178	0.538	0.061632	0.055748
	ANN	0.9989	0.0028	0.9998	0.085	0.257	0.027142	0.024551
	MCR-ALS	1.0349	− 0.0632	0.9981	0.240	0.727	0.098257	0.088886
ANT imp-A	PLS	1.0109	− 0.0025	0.9992	0.093	0.292	0.007856	0.018384
	ANN	1.0000	− 0.0004	1.000	0.001	0.004	0.000437	0.000395
	MCR-ALS	1.0249	− 0.0143	0.9995	0.073	0.222	0.035440	0.032056
PABA	PLS	0.9770	0.0098	0.9994	0.042	0.126	0.002338	0.003565
	ANN	1.0140	− 0.0067	0.9997	0.034	0.102	0.012623	0.011418
	MCR-ALS	0.9719	0.0168	0.9982	0.069	0.209	0.024062	0.021765

^a^LOD = 3.3 σ/ S LOQ = 10 σ/ S where, σ is the SD of the response and it was calculated separately for each component in each model, and S is the slope obtained by drawing calibration curves between the theoretical concentrations and the found concentration for each component using the developed models

^b^SEP = $\sqrt{\frac{\sum ({y}_{r}-{y}_{p}-bais{)}^{2}}{n-1}}$, where y_r_ and y_p_ is the true and predicted values, respectively, n is the number of samples used in validation and bias = $\frac{\sum \left({y}_{r}-{y}_{p}\right)}{n}$, While RMSEP = $\sqrt{\frac{\sum ({y}_{r}-{y}_{p}{)}^{2}}{n}}$

#### Artificial neural network (ANN) model

ANN is a type of emulated intelligence technique consisting of numerous highly interconnected artificial neurons or nodes that can mimic the functioning of a biological nervous system. ANNs are designed to find solutions to complex data problems. Each connection, like the synapses in a biological brain, transmits signals to other neurons. ANN incorporates three layers: input, hidden, and output. Signals travel from the first layer (the input layer) through the hidden layer to the final layer (the output layer) [38, 43]. In our case, various networks were created to identify the optimal neural network model for determining the cited components. The results from the fabricated network were compared to select the best model. The input layer consisted of 326 neurons, corresponding to the number of spectral data points utilized. In contrast, the output layer contained 4 neurons, representing the number of drugs to be quantified in each mixture. The number of neurons in the hidden layer was adjusted based on a trial-and-error approach. As observed, there was a significant decrease in the RMSEC value on increasing the number of the hidden neurons from 2 to 5. However, no significant change was noted when the number of hidden neurons exceeded 5. Additionally, a learning rate of 0.1 and 50-epochs were used. As shown in Table 2, the mean recovery percentages, standard deviations (SDs %), and specificity relevant standard deviations (RSD%) for each component are summarized, demonstrating the successful predictive ability of the ANN model. Table 3 presents the linearity parameters, limits of detection and quantification, as well as the standard error of prediction (SEP), and root mean square error of prediction (RMSEP).

#### Multivariate curve resolution-alternating least squares (MCR-ALS) model

In a bilinear MCR model, the spectral data set D was factorized into concentration “C” and spectra “S”, ensuring that constraints on both C and S are met [44]. The MCR bilinear model is typically expressed as **D** = **CST,** where **D** represents the raw data set (spectroscopic data), while **ST** and **C** are the matrices of the pure spectra and the corresponding concentration profiles for each compound (contribution) in the system. The recorded spectral data matrix is decomposed into the spectral profiles and the concentration matrices of the pure components in the samples; after which, the error is calculated. Since the decomposition of the data matrix does not yield unique solution, the number of possible solutions can be quite large. Therefore, applying constraints such as non-negativity, closure, unimodality, and equality is advantageous in this context [7].

**Fig. 4:**
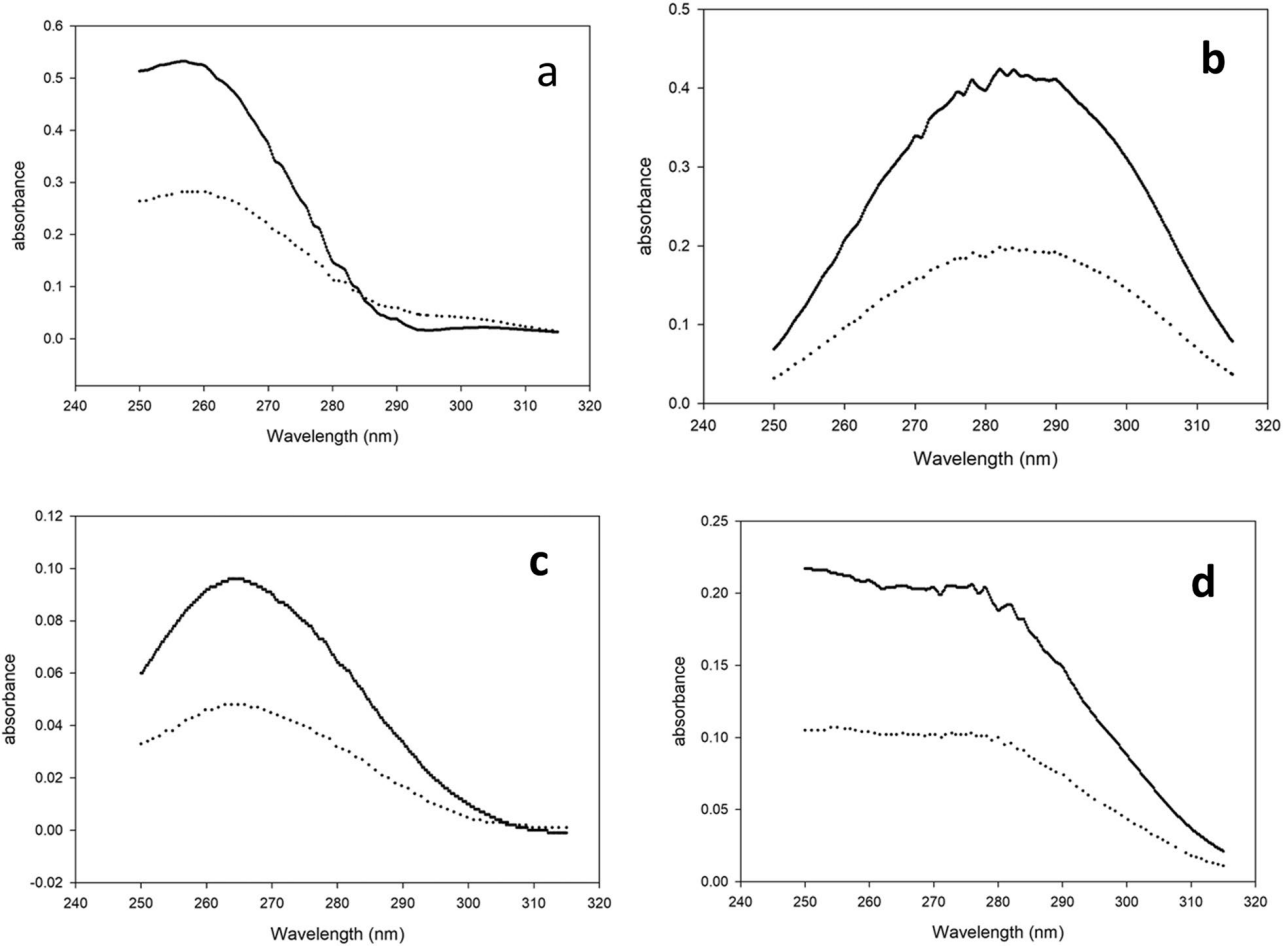
Pure spectra (solid line) and extracted spectra by MCR-ALS (dotted line) for the four cited components **a** ANT, **b** BEN, **c** ANT imp-A, and **d** PABA

In this work, a non-negativity constraint for concentration was applied, to ensure that the concentration values were above or equal to zero. Also, an equality constraint (lower than) was applied for concentration. The ALS optimization process ended after 9 iterations, yielding a calculated percentage of lack of fit of 0.28784 and an r^2^ value of 99.9743. These results were satisfactory and supported the quality of the proposed model. The MCR-ALS model has the advantage of providing qualitative determination within its algorithms, allowing for the evaluation of the spectra of the cited drugs. Figure 4 shows the resemblance of the pure spectra. The recoveries, SDs% and the specificity RSD% for the validation set mixtures are summarized in Table 2. Additionally, all regression parameters, including the LOD, LOQ, SEP, and RMSEP, are presented in Table 3.

The assessment of the three proposed models, revealed that the close values between SEP and RMSEP in all models indicated the absence of overfitting in the constructed models, demonstrating the high prediction ability of each model. However, the ANN model gave more precise results than both the PLS and MCR-ALS, with lower LOD and LOQ. Therefore, the ANN model is preferred for drug quantification over the PLS and MCR-ALS models. However, MCR-ALS has the additional advantage of being capable of conducting both qualitative or quantitative analysis of the analyte.

On the other hand, when comparing our models with the previously reported methods for the analysis of ANT and BEN [17, 45], the published method for ANT determination has the advantage of its wide linearity range. However, this limitation in the developed methods occurred due to the high response of ANT and BEN when analyzed together. Conversely, our study has the advantage of using three chemometric models (PLS, ANN, and MCR-ALS) for the determination of ANT, as opposed to only one model used in the published method. In addition, our method calculated the LOQ for the studied drug. Furthermore, our method successfully determined ANT in the presence of its official impurity, ANT imp-A, which was not considered in the reported one. Finally, greenness and whiteness assessments were performed in our study, which were not addressed in the reported study. Compared to the reported method for BEN which has the advantage of wider linearity range, our method offers several benefits. First, the reported method did not specify the LOD or LOQ for the drug, whereas our method did. Second, the reported method utilized only two models for analyzing BEN, while our study employed three. Third, our method successfully determined the drug in the presence of its degradation product, a feature not included in the reported method. Finally, the reported method did not conduct greenness and whiteness assessments, as demonstrated in Table 4.

**Table 4:** Comparison between the reported chemometric methods for the determination of ANT and BEN and the proposed method

Ref. No	Analyte to determine	Linearity range	LOD	LOQ	Model of determination	Application	Advantage	Disadvantage
[17]	ANT	4.0–20.0 μg mL^−1^	0.3 μg mL^−1^	NA	PLS	- Pharmaceutical dosage form	- Wider linearity range	- LOQ not available - Only one model used
[45]	BEN	2.0–18.0 μg mL^−1^	NA	NA	PLS and PCR	- Pharmaceutical dosage form	- Wide linearity range	- LOD and LOQ not reported
This work	ANT	5.0–9.0 μg mL^−1^	0.312 μg mL^−1^ (PLS), 0.185 μg mL^−1^ (ANN), 0.473 μg mL^−1^ (MCR)	0.945 μg mL^−1^ (PLS), 0.561 μg mL^−1^ (ANN), 1.432 μg mL^−1^ (MCR)	PLS, ANN and MCR-ALS	- Pharmaceutical dosage form - Determination of the two cited drug in presence of their impurity - Green and white profile assessment	- LOD and LOQ calculated - Three models applied - The drugs were determined along with their impurities - Greenness and whiteness assessment performed	- Limited linearity range
	BEN	1.0–5.0 μg mL^−1^	0.178 μg mL^−1^ (PLS), 0.085 μg mL^−1^ (ANN), 0.240 μg mL^−1^ (MCR)	0.538 μg mL^−1^ (PLS), 0.257 μg mL^−1^ (ANN), 0.727 μg mL^−1^ (MCR)				

### Ear drops application

The successful determination of ANT and BEN in ear calm drops was achieved using the constructed multivariate chemometric methods. As shown in Table 5, satisfactory recoveries were obtained, and the validity of the models was evaluated using the standard addition technique.

**Table 5:** Determination of ANT, BEN in their dosage form and application of standard addition technique using the proposed the proposed PLS, ANN and MCR-ALS models

Model	Ear calm® ear drop	% Found Mean^a^ ± SD	Standard addition technique				
			Taken µg mL^−1^	Added µg mL^−1^	Found µg mL^−1^	Recovery %	Mean ± SD
PLS	ANT	97.56 ± 0.070	3.0	1.5	4.45	98.24	98.42 ± 0.894
				3.0	5.97	98.92	
				6.0	8.94	98.09	
	BEN	100.63 ± 0.304	1.0	0.5	1.52	102.08	102.04 ± 1.747
				1.0	2.01	100.74	
				2.0	3.03	103.31	
ANN	ANT	100.79 ± 0.886	3.0	1.5	4.50	100.10	100.47 ± 0.581
				3.0	6.01	100.21	
				6.0	9.03	101.10	
	BEN	100.78 ± 0.838	1.0	0.5	1.50	100.29	100.77 ± 0.836
				1.0	2.01	100.62	
				2.0	3.01	101.41	
MCR-ALS	ANT	103.13 ± 1.823	3.0	1.5	4.54	101.29	101.03 ± 1.928
				3.0	6.08	102.77	
				6.0	8.97	99.04	
	BEN	101.11 ± 1.615	1.0	0.5	1.51	101.34	100.58 ± 1.574
				1.0	1.99	98.84	
				2.0	3.02	101.55	

^a^Average determinations of three ear drops dosage form

### Statistical analysis

Student’s t-test and F- one were statistically calculated to compare the results obtained from our models with those obtained by the reported method for ANT and BEN [22] in their pure forms. The outcomes of this comparison, including mean recoveries, standard deviations, and variances are summarized in Table 6. The results demonstrated no significant statistical differences between the methods.

**Table 6:** Statistical comparison between the results obtained by the proposed PLS and ANN models and the official BP method of analysis of ANT, BEN

Parameter	PLS		ANN		MCR-ALS		Reported method [22]^b^	
	ANT	BEN	ANT	BEN	ANT	BEN	ANT	BEN
Mean	98.66	100.77	100.27	99.99	100.26	100.84	100.03	99.08
SD	1.875	1.974	0.797	1.054	2.095	1.996	0.961	0.854
Variance	3.515	3.895	0.635	1.111	4.389	3.983	0.924	0.729
n	11	11	11	11	11	11	5	5
Student’s t-test	1.529 (2.145)^a^	1.813 (2.145)^a^	0.526 (2.145)^a^	1.692 (2.145)^a^	0.226 (2.145)^a^	1.870 (2.145)^a^	–	–
F-test	3.806 (5.964)^a^	5.342 (5.964)^a^	1.454 (3.478)^a^	1.53 (5.964)^a^	4.752 (5.964)^a^	5.462 (5.964)^a^	–	–

^a^These values represent the corresponding tabulated values of t and F at p = 0.05

^b^ANT and BEN were analyzed using HPLC method using C8 column, acetonitrile: phosphate buffer pH 5.5 (25.0: 75.0, *by volume*) as mobile phase and UV detection at 270.0 nm

### Green profile assessment

**Fig. 5:**
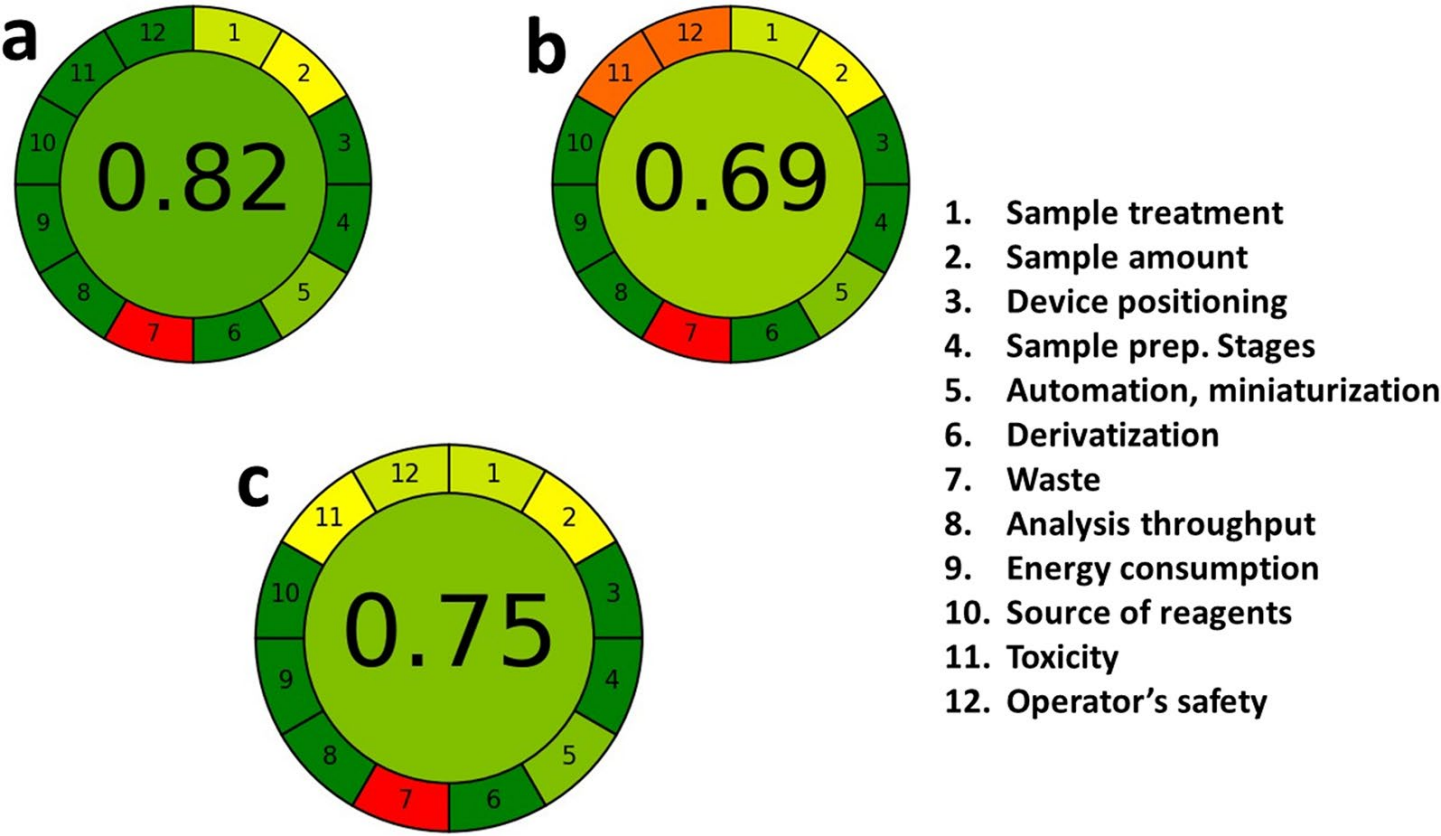
AGREE green profile assessment of **a** the proposed chemometrics method for ANT and BEN determination, **b** the reported chemometrics method for ANT determination and **c** the reported chemometrics method for BEN determination

The greenness of the proposed method was assessed using the green analytical evaluation tools, Eco-scale and AGREE. The Eco-scale tool is a semi-quantitative method that has the advantage of being easy to use for evaluating various environmental aspects [46, 47]. Its assessment depends on the subtraction of penalty points related to the solvent used, energy consumption, waste product, and hazardousness of the used reagent from a base of 100. If the calculated Eco-scale score is more than 75 and close to 100, the method is considered a green method [34, 48]. The Eco-scale score was calculated for the proposed chemometric methods and for the other reported ones [17, 45]. As provided in Table 7**,** the Eco-scale score of the proposed method was found to be 96, which is higher than that of the reported methods, indicating the greenness of the developed methods. AGREE, one of the recent tools for greenness evaluation, is a calculator tool where 12 green analytical chemistry principles were taken into account and converted into a score between 0 and 1 using a pictogram representation [35]. The circle becomes greener when the obtained score is closer to 1. The pictograms of the proposed method and the published method are shown in (Fig. 5). The pictograms of the proposed method scored 0.82, indicating that the greenness of the proposed method superior to the reported methods. Finally, based on the attained results, the proposed method had provided a green analytical profile.

**Table 7:** Greenness evaluation of the proposed and reported chemometrics methods for the determination of ANT and BEN using analytical Eco-scale tools

Proposed method		Reported method [17]		Reported method [45]	
Analytical eco-scale	Penalty point	Analytical eco-scale	Penalty point	Analytical eco-scale	Penalty point
Reagents		Reagents		Reagents	
Distilled water	0	Ethanol	4	0.01 N HCl	4
		0.1 N HCl	4		
Instrument		Instrument		Instrument	
Energy (< 1.5 kWh per sample)	1	Energy (< 1.5 kWh per sample)	1	Energy (< 1.5 kWh per sample)	1
Occupational hazard	0	Occupational hazard	0	Occupational hazard	0
Waste	3	Waste	3	Waste	3
Total penalty points	4	Total penalty points	12	Total penalty points	8
Eco-scale score	96	Eco-scale score	88	Eco-scale score	92

### White profile assessment

**Fig. 6:**
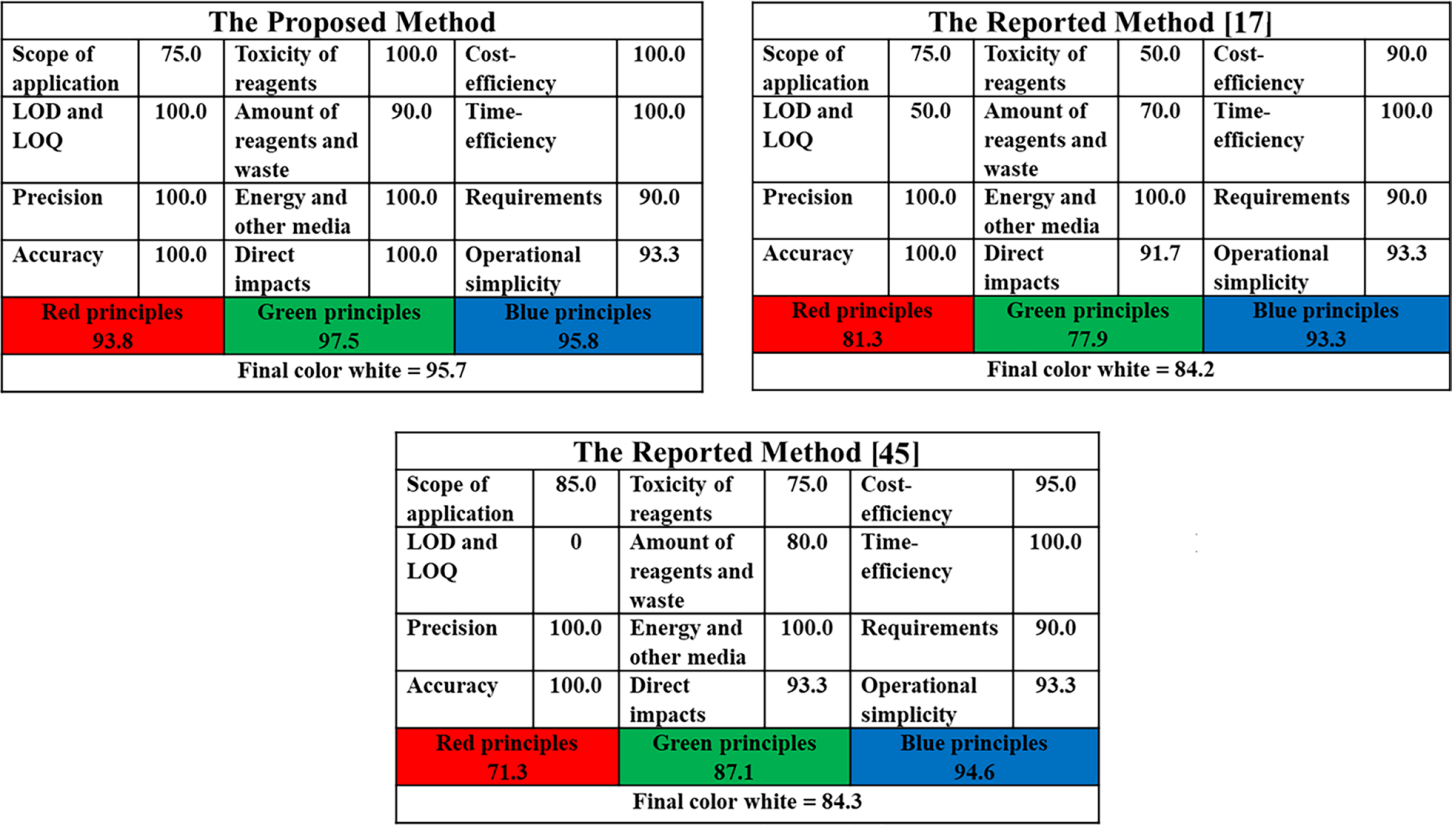
Evaluation of the whiteness of the proposed method and the reported methods according to the RGB12 tool

The RGB model equally distributes the 12 WAC principles into three categories: 4 red principles (R1-R4) concerned with the analytical method, 4 green principles (G1-G4) related to environmental safety, and 4 blue principles (B1-B4) related to economical and practical aspects [36, 49]. After computational assessment, the method gained a color resulting from mixing the previously mentioned colors. The color of the most appropriate analytical method is white, due to the high saturation of each primary color. The whiteness of the proposed method was assessed and compared with the reported methods [17, 45], as shown in (Fig. 6), indicating that the proposed method is regarded as whiter than the other reported methods.

## Conclusion

In this work, ANT and BEN, along with their impurities ANT imp-A and PABA, were quantified in their quaternary mixtures and pharmaceutical dosage forms using simple and accurate chemometrics-assisted spectrophotometric methods. The proposed models included PLS, ANN, and MCR-ALS. The constructed models are considered green, economical and efficient compared to other analytical methods. The greenness and the whiteness of the method was evaluated using analytical Eco-scale, AGREE, and RGB tools. The error was predicted in to be in all models. PLS assumes that the error is equally distributed between the concentration and spectral response; consequently, by excluding the data noise from both the concentration and the absorbance, robust results were achieved using this model. On the other hand, the ANN model’s high predicting ability was utilized to obtain more precise results during the quantification of studied drugs. Furthermore, MCR-ALS provided additional qualitative analysis tools alongside the quantitative one. The extracted spectral profiles of the analyzed drugs showed a strong correlation with their corresponding pure profiles. The proposed methods offer an alternative platform form for the routine analysis of the cited drugs in the pharmaceutical industry.

## Data Availability

The datasets used and/or analysed during the current study available from the corresponding author on reasonable request.

## Abbreviations

AGREE: Analytical Greenness tool
ANT: Antipyrine
ANT imp-A: Antipyrine impurity A
ANN: Artificial neural networks
BEN: Benzocaine HCl
BP: British pharmacopeia
GAC: Green analytical chemistry
LVs: Latent variables
LOD: Limits of detection
LOQ: Limits of quantification
MCR-ALS: Multivariate curve resolution-alternating least squares
PABA: P-amino benzoic acid
PLS: Partial least squares
Ph. Eur.: European pharmacopoeia
USP: United States pharmacopeia
RGB: Red–Green–Blue
RMSEC: Root mean square error of calibration
SEP: Standard error of prediction
WAC: White analytical chemistry
